# Are 3D FSE MRI sequences of the knee at 1.5 T effective in the detection of meniscal and ligament tears? How useful are they?

**DOI:** 10.1590/0100-3984.2016.49.2e1

**Published:** 2016

**Authors:** Artur da Rocha Corrêa Fernandes

**Affiliations:** 1 Associate Professor in the Department of Diagnostic Imaging, Escola Paulista de Medicina da Universidade Federal de São Paulo (EPM-Unifesp), São Paulo, SP, Brazil. E-mail: artur_personal@yahoo.com.br.

The use of fast spin-echo (FSE) magnetic resonance imaging (MRI) sequences is the most versatile way of evaluating the musculoskeletal system. Anisotropic, two-dimensional (2D) sequences have greater resolution in the acquisition plane whereas the voxel resolution in isotropic three-dimensional (3D) volumetric sequences is the same in all planes with no loss of spatial resolution and with no distortion^(1)^.

Initially, 3D gradient-echo sequences which had acceptable acquisition time and high resolution but inadequate tissue contrast were the standard for evaluating the musculoskeletal system^(1-5)^. As of 2007, technological advances led to more consistent use of 3D FSE sequences in such evaluations^(1)^, because 3D FSE sequences provide greater tissue contrast for clinical application in the knee than do 3D gradient-echo sequences^(6)^.

Imaging studies of the knee structures can require the use of oblique planes with special orientation. That is the case of anterior cruciate ligament evaluation, a common situation on the daily practice. In 2007, Rajeswaran et al.^(3)^ highlighted the value of using oblique planes to evaluate the popliteofibular ligament, which plays a role in instability related to lesions of the posterolateral corner structures of the knee.

Evaluations of the articular cartilage of the knee can also benefit from the use of 3D isotropic volumetric sequences due to its capacity to reformat in different planes and its greater spatial resolution which improves the characterization of cartilagineous lesions^(4)^. This last characteristic has a particular application in the femoral trochlea. The articular cartilage evaluation in this area, using axial planes with the usual angulation, has certain limitations. The sagittal plane is called in order to complement this evaluation, although it can be less precise due to partial volume effects^(3)^. Para-axial slices, perpendicular to the articular surface of the trochlea, can better define injury in this area. Regarding the detection of ligament and meniscal injuries, the performance of volumetric sequences is similar to that of 2D sequences, although the quality of 3D images is considered inferior to that of 2D images.^(4,5)^

Although some authors question the real value of 3D FSE sequences for the diagnosis of meniscal and cartilaginous lesions in 1.5 T MRI scanners, those same authors acknowledge their value in providing better characterization of the injuries and improving diagnostic reliability as well as in reducing the total examination time^(7)^. Other researchers, also using 1.5 T scanners, obtained results similar to or even better, in some cases, than those obtained with 2D sequences for the detection of knee injuries^(8)^. In studies employing 3 T scanners, the signal-to-noise ratio for cartilage has been found to be better in 3D FSE sequences which also provided similar or better results for detecting ligament, meniscal, and cartilaginous lesions^(9,10)^, albeit with less specificity^(10)^.

Evaluating meniscal injuries in particular, Kijowski et al.^(11)^ obtained results with 3D FSE sequences that were similar to those obtained with 2D sequences, except in the detection of root tears in the posterior horn of the lateral meniscus, for which the former had lower sensitivity. In addition, 3D FSE sequences can produce blurred images due to greater T2 decay caused by their long echo train^(2)^. However, bone marrow alterations with a pattern of edema and subchondral alterations have been well documented through the use of 3D FSE sequences^(10-12)^.

In this issue of **Radiologia Brasileira**, Chagas-Neto et al.^(13)^ present their findings on the use of FSE volumetric sequences compared with the 2D standard protocol. The great merit of this study is in its employment of such sequences using a 1.5 T scanner which is the MRI equipment most commonly found in Brazil and in many other countries. There have been relatively few studies adopting that approach. In 2012 and 2015, respectively, Ai et al.^(8)^ and Pass et al.^(7)^, both of whom also employed 1.5 T scanners, presented conflicting results regarding the substitution of volumetric sequences for the traditional 2D protocol in knee evaluations. However, both of those studies emphasized the clinical value of 3D sequences in allowing reformatting in any plane and representing a quicker option for patients in pain or who suffer from claustrophobia^(2)^.

In the study conducted by Chagas-Neto et al.^(13)^ there was a short interval between MRI and arthroscopy which is truly difficult to achieve in practice. Although they found the volumetric sequences to be less effective in detecting lateral meniscus tears than in detecting other types of tears, that was true for both techniques and there was good concordance between the two techniques in the evaluation the lateral meniscus. In a similarly designed study, that same group of authors compared volumetric and 2D sequences for the semiquantitative assessment of knee osteoarthritis in 1.5 T scanners and obtained good results^(14)^.

It is important for academic researchers doing high-quality work in Brazil to submit their contributions to this journal (the official organ of the Brazilian College of Radiology and Diagnostic Imaging). It should be borne in mind that in many residency programs, particularly those in Brazilian countryside, where this publication is a valuable training instrument.
